# Validation of the Effectiveness of a Behavioral Activation-Based Digital App for Treatment of Depressive Symptoms: A Randomized Controlled Trial

**DOI:** 10.3390/bs15111496

**Published:** 2025-11-04

**Authors:** Yongjoon So, Jaeeun Shin, Sung-Doo Won, Wooyoung Im, Kwang-Ho Seok, Min Jin Jin, Seung Ho Lee, Sung-Man Bae

**Affiliations:** 1 Department of Psychology, Chung-Ang University, Seoul 06974, Republic of Korea; psyso@cau.ac.kr (Y.S.); rheai@cau.ac.kr (J.S.); 2 Department of Psychology, Daegu Catholic University, Daegu 42472, Republic of Korea; wonfuture@cu.ac.kr; 3 Department of Psychiatry, Konyang University Hospital, College of Medicine, Konyang University, Daejeon 35365, Republic of Korea; imwy@kyuh.ac.kr; 4 School of AI Convergence, Global Cyber University, Cheonan 31228, Republic of Korea; monoskh@gw.global.ac.kr; 5 Division of Liberal Arts, Kongju National University, Gongju 32588, Republic of Korea; jin@kongju.ac.kr; 6 Psychotherapy Institute for Trauma & Attachment, Asan-si 31465, Republic of Korea; magoship@dankook.ac.kr; 7 Department of Psychology, Graduate School, Dankook University, Cheonan 31116, Republic of Korea; 8 Department of Psychology and Psychotherapy, College of Health Science, Dankook University, Cheonan 31116, Republic of Korea

**Keywords:** behavioral activation, mobile health application, depression, young adults, digital intervention

## Abstract

Our research investigated how a smartphone application utilizing behavioral activation principles affects depression levels in young adult populations. A total of 67 participants aged 20–30 years with clinically significant depressive symptoms (CESD-11 ≥ 16) were divided into treatment (n = 31) and comparison conditions (n = 36) through randomization procedures. Participants in the experimental group engaged with a BA-based mobile application (Maummove) over an eight-week period, while those in the control group completed weekly assessments without intervention. Depression, perceived stress, and life satisfaction were measured at baseline and postintervention using the CESD-11, PSS, and SWLS, respectively. The results indicated that the experimental group exhibited significant reductions in depression (Cohen’s d = 1.03) and stress (Cohen’s d = 0.99) compared to the control group, which showed minimal changes. Improvements in life satisfaction were observed in the experimental group, with a smaller effect size (Cohen’s d = 0.23). Time-series analyses demonstrated that depressive symptoms decreased progressively throughout the intervention period, falling below the clinical cutoff by the seventh week. These findings provide preliminary evidence that BA-based mobile applications may offer a promising, accessible approach to reducing depressive symptoms and perceived stress in young adults, though replication in larger samples with longer follow-up periods is needed to establish generalizability. This study highlights the potential of digitally delivered BA interventions as a viable alternative or complement traditional mental health services, particularly for populations facing barriers to face-to-face care.

## 1. Introduction

In recent years, young adults in South Korea have experienced elevated levels of depressive symptoms, which has emerged as a serious social issue. Mental health challenges among young adults, particularly regarding employment and social integration, remain significantly higher in South Korea compared to OECD average levels (OECD, 2021). The global health crisis precipitated by COVID-19 has further exacerbated psychological distress among young adults worldwide, with particularly notable increases in depression indicators. (McGinty et al., 2022).

The main symptoms of depression in young adults include persistent sadness, fatigue, loss of interest and motivation, changes in sleep and appetite, difficulty concentrating, and suicidal ideation (Auerbach et al., 2018). Perceived stress stemming from academic pressure, employment uncertainty, and interpersonal challenges can contribute to the onset of depression (Liu et al., 2024). Furthermore, numerous studies have shown that excessive exposure to digital environments, such as social media and the comparison culture, adversely affects depression and self-esteem among young adults. Recent research has highlighted the intersection of technology work and mental health, with studies showing that empathy significantly affects software practitioners’ well-being (Cerqueira et al., 2024) and that gender disparities in mental health persist throughout the software engineering pipeline (Takaoka et al., 2024). Additionally, research on workplace environments demonstrates that sense of belonging in technology teams significantly impacts mental health outcomes (Trinkenreich et al., 2024).

Depression adversely affects multiple aspects of young adults’ lives. It is closely associated with poor academic performance, difficulties maintaining employment, interpersonal conflicts, and reduced life satisfaction (Brody & Hughes, 2025; Liu et al., 2024; Estrella-Proaño et al., 2024). If left untreated, depression can even escalate to suicidal thoughts or attempts. Furthermore, barriers to accessing mental health services and the stigma surrounding mental illness often prevent young adults from seeking appropriate help (Brenes et al., 2015). These findings underscore the urgent need for early interventions and accessible psychological treatment programs.

Since its emergence in the early 2000s, behavioral activation has gained recognition as an efficacious therapeutic approach for addressing depressive disorders. Unlike cognitive behavioral therapy (CBT), which focuses on both cognitive restructuring and behavioral change, behavioral activation specifically targets the behavioral component of depression without requiring complex cognitive interventions. While cognitive therapy (CT) primarily addresses maladaptive thought patterns, BA operates on the principle that behavioral changes can lead to mood improvements without directly targeting cognitions (Martell et al., 2021). This distinction is particularly important for digital implementation. CBT requires sophisticated cognitive restructuring exercises and complex therapeutic dialogues that are challenging to replicate in automated mobile applications. In contrast, BA’s focus on concrete, observable behaviors—such as activity monitoring, goal setting, and behavioral scheduling—translates more naturally to mobile app interfaces and automated delivery systems. Behavioral activation is an evidence-based treatment that aims to reduce depressive symptoms by decreasing avoidance behaviors and increasing engagement in value-based activities through systematic activity scheduling and monitoring (Martell et al., 2021). Recent empirical evidence suggests that behavioral activation therapy is as effective as medication or cognitive behavioral therapy (CBT) in treating depression (Dimidjian et al., 2006; Ekers et al., 2014). For instance, a study involving 241 patients with major depressive disorder found that behavioral activation was more effective than cognitive therapy, particularly among individuals with severe symptoms (Dimidjian et al., 2006). Similarly, among hospitalized patients, behavioral activation was more effective than standard supportive psychotherapy, resulting in higher patient satisfaction and participation rates (Hopko et al., 2003).

Meta-analyses reported that behavioral activation demonstrated a large effect size in alleviating depressive symptoms, with benefits persisting even after medication discontinuation (Cuijpers et al., 2007; Richards et al., 2016). In a meta-analysis of 26 studies involving 1524 participants, Ekers et al. (2014) found that behavioral activation produced a large effect size (SMD = −0.74) compared to control conditions, with no significant differences from the effects of medication or CBT. Moreover, behavioral activation has been shown to be effective not only for depression but also for managing stress and enhancing overall mental well-being (Stein et al., 2021). For example, a study on college students demonstrated that behavioral activation interventions significantly reduced depressive symptoms associated with academic stress and improved life satisfaction (Kim et al., 2019). A recent comprehensive meta-analysis by Alber et al. (2023) confirmed that internet-based behavioral activation produces moderate to large effect sizes in reducing depressive symptoms, with effects comparable to face-to-face interventions. Recent randomized controlled trials have further demonstrated the efficacy of fully automated mobile-based behavioral activation interventions, with Santopetro et al. (2024) reporting significant improvements in depressive symptoms using text message-based BA approaches.

The main advantages of behavioral activation are its simplicity, ease of application, and cost-effectiveness. Richards et al. (2016) demonstrated that behavioral activation delivered by trained paraprofessionals (individuals who received structured BA training but lack formal mental health credentials) achieved outcomes comparable to CBT delivered by licensed therapists, while reducing costs by approximately 20%. These paraprofessionals typically completed 5–8 days of intensive BA training workshops that covered (1) basic principles of behavioral activation theory; (2) activity monitoring and scheduling techniques; (3) value identification and goal-setting procedures; (4) basic therapeutic communication skills; and (5) crisis recognition and referral protocols. This structured but time-limited training model demonstrates BA’s accessibility for implementation by individuals without extensive mental health education, supporting its feasibility for digital delivery with minimal human oversight. Furthermore, Gawrysiak et al. (2009) demonstrated that brief behavioral activation interventions significantly outperformed active control conditions in reducing depressive symptoms among university students, with effect sizes comparable to longer therapeutic interventions. Unlike other therapeutic approaches that require complex cognitive restructuring, behavioral activation emphasizes increasing engagement in activities that provide positive reinforcement in daily life, rather than attempting to directly control negative thoughts and rumination (Lejuez et al., 2011). These characteristics make behavioral activation particularly suitable for digital implementation, such as through mobile applications (Dahne et al., 2019).

Behavioral activation has also been proven to be effective when delivered online. A meta-analysis of 11 studies involving 2060 participants found that Internet-based behavioral activation produced moderate-to-large effect sizes (g = 0.69) in reducing depressive symptoms among adults and adolescents with mild-to-severe depression, and that its positive effects were comparable to those of other online psychotherapies (Huguet et al., 2018). These findings suggest that the core principles of behavioral activation can be successfully adapted to digital environments. The selection of behavioral activation over CBT for this mobile intervention was based on several key advantages: (1) simplified intervention structure requiring less extensive training, making it suitable for automated delivery; (2) focus on concrete, measurable behaviors rather than abstract cognitive processes, which translates effectively to mobile app interfaces; (3) demonstrated effectiveness comparable to CBT with large effect sizes (Ekers et al., 2014); (4) cost-effectiveness with approximately 20% lower delivery costs than traditional CBT (Richards et al., 2016); and (5) reduced barriers to implementation, as BA can be delivered effectively by trained paraprofessionals or through digital platforms with minimal human oversight. These characteristics make behavioral activation particularly well-suited for scalable mobile health interventions.

Rapid advancements in mobile technology and the COVID-19 pandemic have significantly accelerated the development and adoption of mobile applications for mental health interventions (Wüllner et al., 2024). Mobile mental health applications offer accessible support anytime and anywhere, particularly for individuals who encounter barriers to traditional face-to-face therapy (Dahne et al., 2019). Apps that apply behavioral activation principles have garnered attention as tools to facilitate engagement in positive activities and mood enhancement (Na et al., 2022). Recent studies have expanded the application of BA-based mobile interventions to diverse populations, including smoking cessation (Borrelli et al., 2024), pregnancy-related depression (Vanderkruik et al., 2024), and examining neurobiological mechanisms underlying behavioral activation effectiveness (Jung et al., 2024). Particularly for digitally literate young adults, mobile apps provide a highly accessible and stigma-free method for receiving mental health support (Jeong & Shin, 2023).

The simple structure and clear behavioral guidelines for behavioral activation make it ideal for mobile app delivery. Users can monitor their activities and moods, identify activities associated with positive emotional states, and set concrete goals to increase their engagement in these activities (Dahne et al., 2019). Moreover, behavioral activation apps can enhance treatment effectiveness by providing real-time feedback, reminders, and reinforcing a sense of accomplishment through goal attainment (Na et al., 2022). Thus, the combination of behavioral activation and digital technology offers an innovative approach to managing depression and stress among young adults.

Despite the growing body of research on mobile mental health interventions, few studies have specifically validated the effects of mobile applications that incorporate behavioral activation principles on depression and stress among young adults. Prior research has largely focused on the general effectiveness of depression apps (Linardon et al., 2019) or user perceptions of technology-based psychological services (Jeong & Shin, 2023), rather than on apps grounded explicitly in behavioral activation theory.

Therefore, this study aimed to verify the efficacy of a mobile application, Maummove, which was developed based on behavioral activation principles, in reducing depressive symptoms and stress among young adults. The findings of this study may serve as foundational evidence for developing accessible digital interventions to address the growing mental health challenges faced by young adults and offer an effective alternative for individuals with limited access to traditional treatments.

## 2. Methods

### 2.1. Participants

This study targeted young adults aged 20–30 years residing in South Korea. The 20–30 age range was selected to target emerging adults, a developmental period characterized by identity exploration and life transitions that create unique stressors and intervention opportunities. This demographic was chosen because (1) they experience elevated depression rates while showing low utilization of traditional mental health services; (2) they demonstrate high smartphone usage and comfort with digital health technologies; (3) this age range corresponds to a relatively homogeneous developmental stage, reducing confounding variables; and (4) the recruitment feasibility in university and community settings was optimal for this demographic.

Participants were recruited through advertisements posted on bulletin boards at six universities, university-affiliated social media groups (Facebook, Instagram), general online communities (Naver Cafe, KakaoTalk open chatrooms), and university websites. Recruitment advertisements were sponsored by the Department of Psychology, Dankook University, and targeted individuals aged 20–30 regardless of student status or employment. During the recruitment process, a preliminary screening questionnaire was administered to assess psychiatric history, suicide risk, alcohol and substance abuse history, and presence of severe physical illnesses to ensure eligibility for participation. Participants were excluded if they met any of the following criteria: (1) current participation in psychotherapy, counseling, or other structured mental health treatment; (2) initiation or dose changes of psychotropic medications within the past 8 weeks; (3) self-reported history of bipolar disorder, psychotic disorders, or active substance use disorders; (4) active suicidal ideation requiring immediate clinical intervention (assessed through screening questions); (5) severe cognitive impairment or physical disabilities that would interfere with smartphone operation; (6) insufficient Korean language proficiency to understand app instructions and assessment materials; and (7) participation in other research studies involving psychological interventions within the past 3 months.

Even after enrollment, participants could withdraw from the study if they voluntarily expressed a desire to discontinue or were unable to use a smartphone or the Internet. Additionally, participants were excluded from analysis if the quality of their collected data was deemed insufficient for analysis due to incomplete responses or protocol non-adherence. All study procedures, including participant recruitment and data collection, were reviewed and approved by the Institutional Review Board of Dankook University (approval no. DKU 2024-09-022-003).

This study was not pre-registered prior to participant enrollment. According to the Clinical Research Information Service (CRIS) guidelines in South Korea, clinical trial registration is currently recommended but not legally mandatory, except for projects designated and funded by the Ministry of Health and Welfare (MOHW), for which registration is compulsory under Article 26-2 of the “Regulations on the Management of Health and Medical Technology Research and Development Projects.” As this study was funded by the National Research Foundation of Korea (NRF) rather than MOHW, pre-registration was not legally required at the time of study initiation (CRIS FAQ: https://cris.nih.go.kr/cris/board/listFAQBoard.do?board_seq=8, accessed on 1 October 2025). The study was retrospectively registered with the Clinical Research Information Service (CRIS registration number: KCT0010928).

During recruitment, depressive symptoms of potential participants were assessed using the Center for Epidemiologic Studies Depression Scale-Short Form (CESD-11) (Radloff, 1977). Individuals scoring 16 or higher on the CESD-11 were eligible for participation and randomly assigned to either the experimental group (treatment mode) or control group. The CESD-11 cutoff score of ≥16 was selected following established research protocols for depression intervention studies (Kohout et al., 1993). This threshold has been consistently used in behavioral activation research with young adults and represents clinically meaningful depressive symptomatology appropriate for psychological intervention while ensuring participant safety.

A total of 312 individuals responded to recruitment advertisements and completed initial online screening. Of these, 89 (28.5%) met the CESD-11 cutoff score of ≥16 and were deemed eligible based on depression criteria. Following comprehensive eligibility screening and informed consent procedures, 78 participants (87.6% of eligible) agreed to participate and were randomized to experimental (n = 42) or control (n = 36) groups. The final analyzed sample comprised 67 participants (85.9% of randomized), with 11 participants from the experimental group excluded due to insufficient app engagement (n = 7) or incomplete assessment data (n = 4).

**Figure 1:**
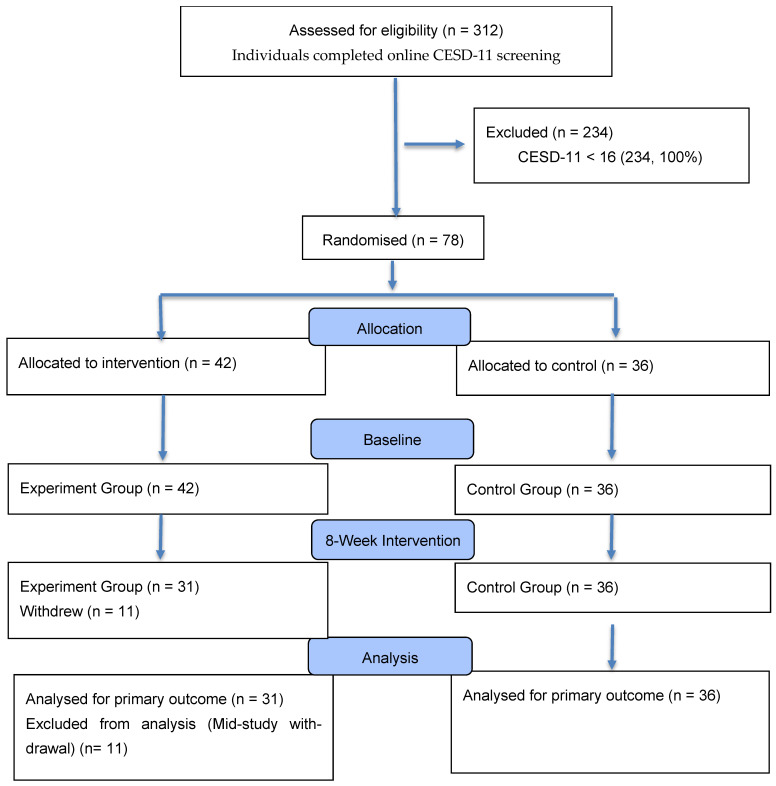
CONSORT flow diagram showing participant recruitment, eligibility screening, randomization, and retention through the 8-week intervention period.

Of the 42 participants assigned to the experimental group, 31 were included in the analysis. All the 36 participants assigned to the control group were included in the final analysis. In the experimental group, there were 5 (16.1%) male and 26 (83.9%) female participants, with a mean age of 27.6 years (SD = 5.69). In the control group, there were 8 (22.2%) male and 28 (77.8%) female participants, with a mean age of 25.5 years (SD = 4.09). Educational composition was as follows: the experimental group had 2 (6.5%) high school graduates, 13 (41.9%) university students, and 16 (51.6%) university graduates; the control group had 3 (8.3%) high school graduates, 19 (52.8%) university students, and 14 (38.9%) university graduates (Figure 1).

### 2.2. Measurements

#### 2.2.1. Depression: Center for Epidemiologic Studies Depression Scale (CESD-11)

Depressive symptoms were measured using the CESD-11, a brief 11-item version of the original CES-D, developed by Radloff (1977) and subsequently validated by Kohout et al. (1993). Participants rated how frequently they experienced each symptom during the past week using a four-point Likert scale: 0 = “Rarely or none of the time (less than 1 day),” 1 = “Some or a little of the time (1–2 days),” 2 = “Occasionally or a moderate amount of the time (3–4 days),” and 3 = “Most or all of the time (5–7 days).” Two items were reverse coded. Higher total scores indicate more severe depressive symptoms, with a score of 16 or higher suggesting probable depression. In this study, the CESD-11 demonstrated excellent internal consistency (Cronbach’s α = 0.926). The CESD-11 was selected over alternatives such as the Beck Depression Inventory (BDI-II) due to its specific design for community populations rather than clinical samples, superior sensitivity to behavioral symptoms of depression that align with behavioral activation principles, and established validity in young adult samples.

#### 2.2.2. Stress: Korean Version of the Perceived Stress Scale (PSS)

Stress levels were assessed using the Korean adaptation of the Perceived Stress Scale (PSS) originally developed by S. Cohen et al. (1983) and validated by Park and Seo (2010). It consists of 10 items rated on a five-point Likert scale (0 = “Never” to 4 = “Very often”), measuring the frequency of perceived stress experiences during the past month. In this study, the Korean version of the PSS demonstrated good internal consistency (Cronbach’s α = 0.892). The PSS was chosen over alternative stress measures due to its focus on perceived rather than objective stress, which better captures the subjective experience targeted by behavioral activation interventions, and its established reliability in university student populations.

#### 2.2.3. Life Satisfaction: Korean Version of the Satisfaction with Life Scale (SWLS)

Life satisfaction was measured using the Satisfaction with Life Scale (SWLS) developed by Diener et al. (1985) and adapted for the Korean population by Suh (1994). The SWLS comprises five items reflecting a global cognitive judgment of an individual’s satisfaction with life, rated on a seven-point Likert scale (1 = “Strongly disagree” to 7 = “Strongly agree”). In this study, the SWLS showed excellent internal consistency (Cronbach’s α = 0.970). The SWLS was selected for its global assessment of life satisfaction, which captures the broader quality of life improvements expected from value-based behavioral activation beyond symptom reduction, and its brief format that minimizes participant burden while maintaining psychometric strength.

### 2.3. Procedure

Recruitment advertisements were posted online, targeting university students and working young adults. Interested individuals completed the online CESD-11 screening questionnaire. Those who scored 16 or higher were provided with detailed information about the study and asked to confirm their willingness to participate. Before beginning the study procedures, all participants were informed that both experimental and control group members would receive modest monetary compensation upon completion of all study requirements, regardless of their level of engagement with the intervention materials. Randomization: Eligible participants (n = 78) were randomly assigned using computer-generated random numbers (Microsoft Excel RAND() function) that allocated either ‘1’ or ‘2’ to each participant with equal probability. A third-party research assistant, independent from recruitment and screening processes, matched these assignments to experimental and control conditions and explained procedures to participants accordingly. This approach ensured allocation concealment as enrollment personnel could not predict upcoming assignments. Final allocation: Experimental n = 42, control n = 36. The experimental group attended an orientation session and subsequently received a link to download the Maummove application, along with a user guide via email. The participants installed the app on their smartphones and engaged in the intervention program over an eight-week period. The Maummove application was developed in Korean language specifically for this study. The app development process followed established behavioral activation principles and mobile health design guidelines, with user interface and content adapted for Korean cultural context.

#### The Maummove Treatment Program Consisted of Two Stages

Stage 1 (Weeks 1–5): The participants monitored their daily activities and emotional states three times per day during the first week to recognize the patterns between behavior and emotion. In Weeks 2–5, the participants identified a value that they deemed important but felt dissatisfied with, engaged in associated positive activities, and evaluated changes in satisfaction at the end of Stage 1.

Stage 2 (Weeks 6–8): Participants reevaluated their values, selected a value with low satisfaction, and planned specific goal-directed activities aligned with this value to enhance their mood and engagement.

The control group also participated in an OT session and received weekly online assessments via Google Forms, mirroring the assessment structure of the Maummove app. Weekly assessments were available from Sunday to Monday evening (10 pm) and missing participants were allowed to complete the assessment the following week.

Upon completion of the eight-week intervention period, participants in both groups were required to complete a post-intervention survey. Participants in the experimental group submitted screenshots of their weekly app usage, and all participation records were stored in a secure database. Both groups received compensation after completing all the study procedures (Table 1).

### 2.4. Analysis

Descriptive statistics were computed to summarize the demographic characteristics (age and sex) of the participants in the experimental and control groups. Second, the pre- and post-intervention scores for depression, stress, and life satisfaction were compared. Means and standard deviations were calculated for each group pre- and post-test. Third, effect sizes (Cohen’s d) were calculated to evaluate the magnitude of change in depression, stress, and life satisfaction within each group from pre- to post-test. Following J. Cohen’s (1988) guidelines, d values of 0.2, 0.5, and 0.8 were interpreted as small, medium, and large effects, respectively.

### 2.5. Power Analysis

No formal a priori power analysis was conducted to determine sample size, representing a methodological limitation. Post hoc power analysis using G*Power 3.1.9.7 revealed that with our achieved sample size (n = 31 experimental, n = 36 control), we had 99.9% power to detect the observed large effect size for depression (d = 1.03) with α = 0.05 using a two-tailed independent samples *t*-test. For a more conservative medium effect size (d = 0.5), our sample provided 70% power. Future studies should conduct prospective power analyses based on expected effect sizes from the previous literature to ensure adequate sample sizes.

## 3. Results

**Figure 2:**
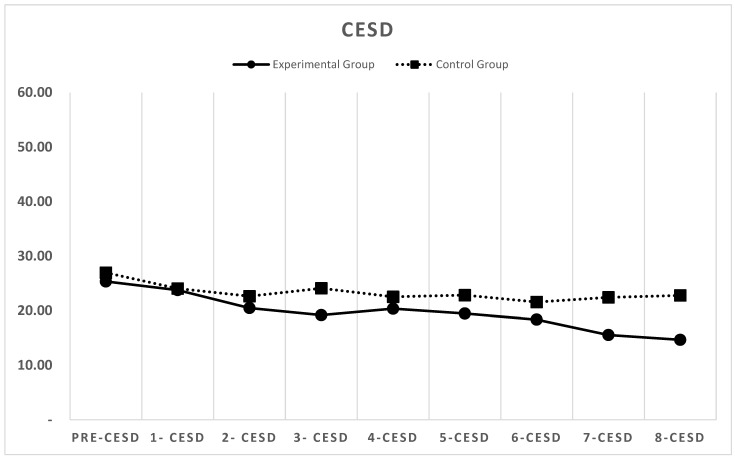
Changes in CESD-11 depression scores for the experimental and control groups across eight weeks.

**Figure 3:**
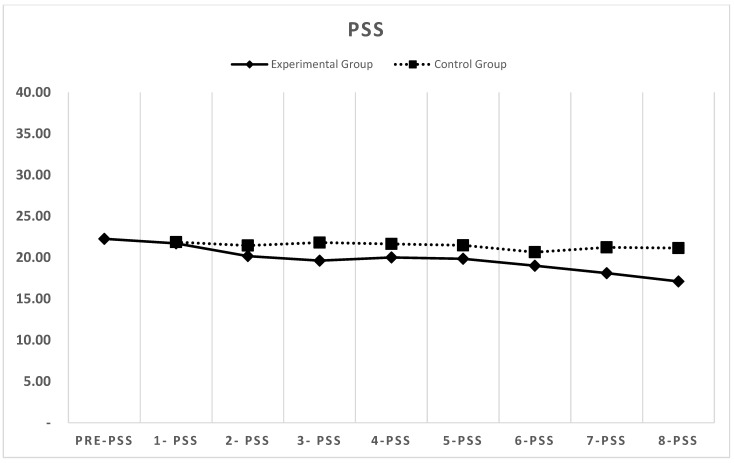
Changes in PSS stress scores for the experimental and control groups across eight weeks.

**Figure 4:**
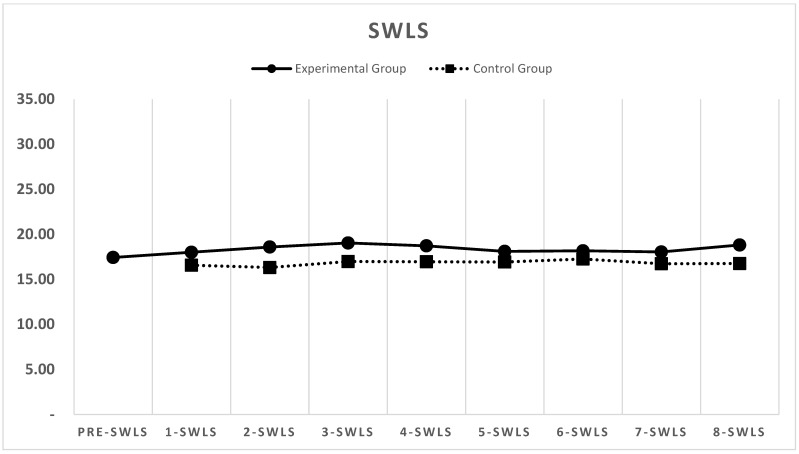
Changes in SWLS life satisfaction scores for the experimental and control groups across eight weeks.

Figure 2, Figure 3 and Figure 4 depict the changes in the depression (CESD-11), stress (PSS), and life satisfaction (SWLS) scores over the eight-week period for both the experimental and control groups (detailed weekly scores are provided in Tables S1–S3 in the Supplementary Materials).

### 3.1. Depression

In the experimental group, the scores for depression decreased significantly over the course of the intervention. The mean pre-test CESD-11 score was 25.34, which decreased to 23.75 after the first week, was below 20 by the third week, and declined below the clinical cutoff score of 16 by the seventh week. By the end of the eighth week, the mean score had further declined to 14.66.

In contrast, no significant changes were observed in the control group. The mean pre-test score was 26.94, and it remained above 22 throughout the study, with a mean score of 22.78 in the eighth week.

Repeated measures ANOVA revealed a significant group × time interaction for depression scores, *F* (8, 520) = 12.47, *p* < 0.001, partial η^2^ = 0.16, indicating differential change patterns between groups over time. Post hoc independent samples *t*-tests showed significant between-group differences beginning at Week 3 (*t* (65) = 2.34, *p* = 0.022, *d* = 0.57) and reaching maximum separation at Week 8 (*t* (65) = 4.78, *p* < 0.001, *d* = 1.24).

Within the experimental group, paired samples *t*-tests revealed significant reductions from baseline at each time point after Week 2 (all *p* < 0.05), with the largest reduction observed from baseline to Week 8 (*t* (30) = 5.67, *p* < 0.001, 95% CI [6.8, 14.6]).

### 3.2. Perceived Stress

Similarly, stress scores in the experimental group progressively decreased throughout the intervention. The mean pre-test PSS score was 22.26, decreasing to 21.71 after the first week and steadily declining thereafter. By the fifth week, the score was below 20, and by the eighth week, it had declined to 17.10.

In contrast, the stress scores in the control group showed minimal fluctuations, with no statistically significant changes. The mean stress score remained at approximately 21 throughout the eight-week period, with a score of 21.14 in the final week.

Mixed-effects ANOVA for stress scores showed a significant group × time interaction, *F* (7, 455) = 8.93, *p* < 0.001, partial η^2^ = 0.12. Between-group comparisons using independent *t*-tests revealed significant differences from Week 4 onward (Week 4: *t* (65) = 1.89, *p* = 0.048; Week 8: *t* (65) = 3.92, *p* < 0.001).

Within-group analysis for the experimental group showed progressive stress reduction with significant decreases from baseline beginning at Week 3 (*t* (30) = 2.41, *p* = 0.022) and maintaining significance through Week 8 (*t* (30) = 4.23, *p* < 0.001, 95% CI [2.8, 7.4]).

### 3.3. Life Satisfaction

Life satisfaction scores changed only modestly in the experimental group. The mean SWLS score at pre-test was 17.42, increasing slightly to 18.00 in Week 1. Although there were minor fluctuations, no substantial trends were observed throughout the intervention period. In the eighth week, the score had increased to 18.81.

Similarly, the life satisfaction scores in the control group remained relatively stable, with minimal changes throughout the study period.

### 3.4. Effectiveness of the Behavioral Activation-Based Application

Effect sizes (Cohen’s d) were calculated to assess the magnitude of change within groups from pre- to post-test and between intermediate time points.

Comparing Week 1 to Week 8 changes, the experimental group showed large effects for depression (d = 0.87) and stress (d = 0.88), while the control group showed negligible effects for depression (d = 0.09) and stress (d = 0.13).

Stress showed a small effect from pre-test to Week 1 (d = 0.10), a large effect from pre-test to Week 8 (d = 0.99), and a large effect from Week 1 to Week 8 (d = 0.88). Life satisfaction showed small effects throughout (d = 0.10 to 0.23).

The large effect sizes observed for depression (d = 1.03, 95% CI [0.61, 1.45]) and stress (d = 0.99, 95% CI [0.57, 1.41]) exceed Cohen’s conventional benchmarks for large effects (d ≥ 0.80) and represent substantial clinical change. These effect magnitudes are comparable to those reported in face-to-face behavioral activation interventions (Ekers et al., 2014; d = 0.74) and suggest clinically meaningful symptom reduction beyond statistical significance. The small effect size for life satisfaction (d = 0.23, 95% CI [−0.17, 0.63]) indicates modest improvement that may require longer intervention periods or additional components to achieve larger changes (Table 2).

In contrast, the control group exhibited negligible effect sizes across all variables, indicating minimal changes throughout the intervention period (Table 3).

#### Conservative ITT-Style Sensitivity Analysis

To examine the robustness of findings despite the unavailability of withdrawn participants’ data, we conducted a conservative sensitivity analysis assuming zero improvement (Week 8 = baseline) for all 11 withdrawn experimental group participants. Under this worst-case assumption, effect sizes remained in the moderate-to-large range and statistically significant.

Depression (CESD-11): Cohen’s d = 0.69, 95% CI [0.28, 1.10], F(8, 608) = 8.42, *p* < 0.001, representing a 33% reduction from the Per-Protocol effect size (d = 1.03) but maintaining clinical significance.

Stress (PSS): Cohen’s d = 0.64, 95% CI [0.23, 1.05], F(7, 532) = 6.15, *p* < 0.001, representing a 35% reduction from the Per-Protocol effect size (d = 0.99) but maintaining clinical significance.

## 4. Discussion

This study aimed to evaluate the effectiveness of a mobile app that applies the principles of behavioral activation to improve mental health, particularly by reducing depressive symptoms in young adults.

The findings demonstrated that the behavioral activation-based mobile application demonstrated meaningful reductions in depression and stress among participants in the experimental group compared to those in the control group. The experimental group demonstrated clinically meaningful reductions in depressive symptoms, with scores falling below established clinical thresholds by the intervention’s conclusion. This pattern supports the theoretical foundation of behavioral activation, which posits that systematic engagement in value-based activities leads to mood improvement through increased positive reinforcement and reduced behavioral avoidance. By contrast, the control group only exhibited a minor decrease in depressive symptoms (Cohen’s d = 0.33). Similarly, stress levels in the experimental group showed a significant reduction (Cohen’s d = 0.99), whereas no significant changes were observed in the control group.

Time-series analyses revealed that improvements in depression and stress demonstrated overall downward trends with notable week-to-week variations throughout the eight-week intervention period, with notable week-to-week variations alongside the overall downward trajectory. Notably, the depression scores fell below 20 by the third week and declined below the clinical threshold by the seventh week, suggesting the early and sustained efficacy of the intervention.

Although the life satisfaction scores increased slightly in the experimental group, the effect size was relatively small (Cohen’s d = 0.23), indicating that more intensive or longer interventions may be required to achieve substantial improvements in overall life satisfaction.

This study makes several important contributions to the field of digital mental health interventions.

First, this is among the first studies conducted in South Korea to empirically validate the effectiveness of a behavioral activation-based mobile application. The results provide preliminary evidence suggesting that the core mechanisms of behavioral activation can be successfully adapted to mobile platforms, supporting the integration of evidence-based psychotherapy principles with emerging digital technologies.

Second, this study provides systematic evidence for the effectiveness of digital mental health interventions specifically targeting young adults, a population increasingly vulnerable to depression and stress but often reluctant to seek face-to-face services owing to stigma and accessibility barriers. While previous studies have examined general mental health apps and user perceptions (e.g., Linardon et al., 2019; Jeong & Shin, 2023), this study uniquely focused on a theoretically grounded, behavioral activation-based intervention.

Third, the results align with and extend the findings from meta-analyses of Internet-based behavioral activation (e.g., Huguet et al., 2018) and mobile mental health interventions (e.g., Torous et al., 2020; Firth et al., 2017). The large effect sizes observed for depression and stress suggest that structured behavioral activation principles retain their efficacy even when delivered via mobile applications, thereby supporting the broader applicability of behavioral activation across diverse digital contexts.

Furthermore, the practical advantages of mobile interventions, such as accessibility, scalability, and cost-effectiveness, are highlighted. Mobile apps offer a stigma-free, flexible alternative to traditional psychotherapy, particularly benefiting digitally fluent populations, such as young adults (Mohr et al., 2018; Fleming et al., 2019).

Finally, this study underscores the potential of structured behavioral interventions to drive real-world behavioral changes. The observed increases in engagement with value-based activities over the intervention period suggest that mobile applications can function not only as monitoring tools but also as catalysts for meaningful behavioral changes (Na et al., 2022; Schueller et al., 2017).

Despite its strengths, this study had several limitations that should be acknowledged: First, the sample size was relatively small (31 and 36 in the experimental and control groups, respectively) with a high proportion of female participants (83.9% and 77.8%, respectively). This demographic skew limits the generalizability of the findings, particularly across sexes. Future studies should recruit more balanced and diverse samples.

Second, the study relied exclusively on self-report measures, which may be subject to biases, such as social desirability and recall inaccuracies. The lack of objective behavioral or physiological data (e.g., app usage logs and passive sensor data) limits the ability to validate self-reported improvements (Baumeister et al., 2019).

Additionally, the passive control condition (weekly assessments only) may have led to overestimation of intervention effects due to attention bias and differential expectancy effects. Participants in the experimental group received structured intervention content and app-based engagement, while controls only completed assessments without any intervention components. An active control condition (e.g., a mood-tracking app without behavioral activation components or a waitlist control with equivalent attention) would have provided a more rigorous comparison by controlling for attention, technology use, and expectancy effects. This design choice was made due to resource constraints and represents a significant limitation that should be addressed in future studies through active control comparisons.

Third, the intervention period was relatively short (eight weeks). Although significant short-term improvements were observed, the long-term sustainability of these effects remains unclear. Prior research (Gilbody et al., 2015) suggests that the effects of digital interventions may diminish over time without continued engagement.

Additionally, the absence of post-intervention follow-up data represents a critical limitation that precludes any conclusions about the sustainability of observed benefits. The 8-week intervention period only demonstrates short-term effectiveness, and it remains unknown whether improvements in depression and stress persist beyond the active intervention period. This is particularly crucial for digital mental health interventions, where engagement typically declines over time and benefits may diminish without ongoing support or booster sessions. Future research must include systematic follow-up assessments at 3, 6, and 12 months post-intervention to establish the durability of effects and inform evidence-based recommendations for maintenance strategies, booster interventions, or transition to traditional care when appropriate.

Fourth, the cultural specificity of our sample significantly limits generalizability. This study was conducted exclusively with Korean young adults using Korean-language materials, and several cultural factors may influence intervention effectiveness: (1) collectivistic cultural values that may affect individual-focused behavioral interventions; (2) cultural stigma around mental health help-seeking that could influence engagement patterns; (3) technology adoption and usage patterns that vary across cultures; and (4) cultural conceptualizations of depression and stress that may differ from Western models underlying behavioral activation theory.

Additionally, our sample was predominantly university-educated (83.9%), limiting applicability to young adults with different educational backgrounds, socioeconomic status, or employment situations. The generalizability of other cultural contexts, healthcare systems, and demographic groups requires empirical investigation through cross-cultural replication studies.

Fifth, the inability to conduct traditional Intention-to-Treat (ITT) analysis represents a methodological limitation. Our IRB-approved ethical protocol required immediate and complete deletion of all data upon participant withdrawal, precluding traditional ITT analysis with imputation methods that require retained data. The differential withdrawal rates between experimental (26.2%, 11/42) and control (0%, 0/36) groups warrant careful consideration. To address this limitation, we conducted a conservative sensitivity analysis assuming zero improvement for withdrawn participants—a worst-case scenario that substantially underestimates intervention effects if withdrawn participants experienced any benefit. Even under this unfavorable assumption, effects remained moderate-to-large (d = 0.69 for depression, d = 0.64 for stress), providing confidence that our conclusions are robust. The true intervention effects likely fall between our conservative estimate and the Per-Protocol estimate, as it is implausible that all withdrawn participants experienced absolutely no improvement.

Sixth, this study was not pre-registered in a public registry (e.g., ClinicalTrials.gov, OSF) prior to data collection, which limits transparency regarding potential selective reporting of outcomes and post hoc analytical decisions. This represents a significant methodological limitation that should be addressed in future research through prospective trial registration with clearly defined primary and secondary outcomes.

Finally, we did not systematically collect detailed participant engagement metrics beyond intervention completion rates. While participants in both groups completed their respective protocols (experimental group: 8-week app usage; control group: 8-week assessments), we did not extract quantitative data on session frequency, duration, or specific feature utilization from the app. This limits our ability to assess dose–response relationships and provide detailed feasibility metrics that would be valuable for future intervention optimization and implementation research.

Building on the current findings, the following directions for future research are outlined.

First, studies with larger and more diverse samples are needed to enhance the external validity of the findings. In particular, analyzing potential differences in intervention effectiveness by sex, age, and socioeconomic status would provide valuable insights into tailoring interventions (Torous et al., 2020).

Second, the application of behavioral activation principles could be extended to address other mental health challenges, such as anxiety and social isolation, in which behavioral avoidance plays a significant role (Carl et al., 2020).

Third, the effectiveness of blended care models, for example, integrating mobile app interventions with face-to-face therapy, should be explored. Such hybrid approaches could maximize treatment engagement and effectiveness while optimizing resource use (Wentzel et al., 2016; Kooistra et al., 2019).

Fourth, Future studies should incorporate comprehensive engagement analytics, including session frequency, duration, feature utilization patterns, and user pathway analysis. Such metrics would enable examination of dose–response relationships, identification of optimal engagement thresholds, and development of personalized intervention algorithms to maximize effectiveness while minimizing participant burden.

Finally, incorporating artificial intelligence (AI) techniques to personalize intervention content and feedback could enhance user engagement and long-term outcomes. Recent advances suggest that AI-driven adaptive interventions have strong potential to improve mental health care delivery (Colombo et al., 2019).

## 5. Conclusions

This study provides preliminary evidence supporting the potential effectiveness of behavioral activation-based mobile applications for reducing depressive symptoms and perceived stress among Korean young adults. The observed effect sizes (depression: d = 1.03; stress: d = 0.99) suggest clinically meaningful improvements that warrant further investigation in larger, more diverse samples.

The progressive pattern of symptom reduction over eight weeks supports the theoretical mechanisms underlying behavioral Activation and demonstrates the feasibility of delivering structured psychological interventions through mobile platforms. The intervention’s effectiveness in achieving clinically significant depression reduction (below CESD-11 cutoff) by Week 7 suggests practical clinical utility.

These findings contribute to the growing evidence base for digital mental health interventions, particularly for young adults who face barriers to traditional mental health services. The accessibility and scalability of mobile-delivered behavioral activation may address treatment gaps in underserved populations, though cultural adaptation and broader validation remain essential.

Future research should prioritize (1) large-scale randomized controlled trials with diverse demographic and cultural groups; (2) longer-term follow-up studies to assess intervention sustainability; (3) cross-cultural validation and adaptation studies; (4) optimization research examining which intervention components drive effectiveness; (5) integration studies combining mobile interventions with traditional care models; and (6) implementation research addressing real-world deployment challenges.

The modest sample size, short intervention duration, cultural specificity, and lack of long-term follow-up necessitate cautious interpretation of these findings. While promising, these results require replication and extension before broader clinical implementation can be recommended.

This study represents an important step toward understanding the potential of technology-enhanced behavioral activation interventions while highlighting the rigorous research agenda needed to establish their clinical utility and optimal implementation approaches.

## Figures and Tables

**Table 1:** Main process for the Maummove intervention group.

Step	Week	Weekly Process
1	1	During the monitoring week, participants recorded their daily activities and emotions three times a day to confirm that their behaviors and emotions were connected.
	2	① At the start of Week 2, participants completed a “Value Understanding” exercise, evaluating the importance and satisfaction levels of eight values: family relationships, marital/romantic relationships, friendships/acquaintances, leisure/hobbies, self-development/growth, work/career/education, spirituality/religion, and health. ② Participants identified one value that was important to them but with which they were dissatisfied. ③ Participants then planned and carried out one pleasant activity related to that value.
	3	Participants planned and carried out one or two pleasant activities related to their values.
	4	Participants planned and carried out up to three pleasant activities related to their values.
	5	Participants planned and carried out up to three pleasant activities related to their values.
2	6	④ At the beginning of Week 6, they reevaluated values to confirm values that were important but had low satisfaction at the time. ⑤ Planned and performed one goal action that aligned well with the values that were important but had low satisfaction and could improve depression.
	7	They planned and carried out 1–2 goal actions that aligned with the relevant values.
	8	They planned and carried out up to three goal actions.

**Table 2:** Effect sizes (Cohen’s d) for changes within the experimental group.

Variable	Week 1 *M* (*SD*)	Week 8 *M* (*SD*)	Week 1–Week 8 *d*
depression (CESD)	23.75 (11.85)	14.66 (8.96)	0.87
Stress (PSS)	21.71 (5.29)	17.10 (5.15)	0.88
Life Satisfaction (SWLS)	18.00 (6.43)	18.81 (6.31)	0.13

Notes. Cohen’s d = 0.2 (small effect size), 0.5 (medium effect size), 0.8 (large effect size).

**Table 3:** Effect sizes (Cohen’s d) for changes within the control group.

Variable	Week 1 *M* (*SD*)	Week 8 *M* (*SD*)	Week 1–Week 8 *d*
depression (CESD)	24.04 (13.12)	22.78 (15.75)	0.09
Stress (PSS)	21.86 (6.08)	21.14 (5.15)	0.13
Life Satisfaction (SWLS)	16.56 (6.47)	16.75 (7.59)	0.03

Notes. Cohen’s d = 0.2 (small effect size), 0.5 (medium effect size), 0.8 (large effect size).

## Data Availability

The datasets generated and/or analysed during the current study are not publicly available but are available from the corresponding author on reasonable request.

## Supplementary Materials

The following supporting information can be downloaded at: https://www.mdpi.com/article/10.3390/bs15111496/s1, Table S1: Depression scores (CESD-11) across eight weeks for the experimental and control groups; Table S2: Stress scores (PSS) across eight weeks for the experimental and control groups; Table S3: Life satisfaction scores (SWLS) across eight weeks for the experimental and control groups.
