# Supplementary material for: Validation of the Effectiveness of a Behavioral Activation-Based Digital App for Treatment of Depressive Symptoms: A Randomized Controlled Trial
Source: Behav Sci (Basel). 2025 Nov 4;15(11):1496. doi: 10.3390/bs15111496 (PMC12649501; doi:10.3390/bs15111496)
Supplement: Supplementary file 1 [file behavsci-15-01496-s001.zip › behavsci-3780505-supplementary.pdf]

behavioral sciences

Supplementary data

Table S1. Depression scores (CESD-11) across eight weeks for the experimental and control groups.

Group	Pretest	Week 1	Week 2	Week 3	Week 4	Week 5	Week 6	Week 7	Week 8
Experimental	25.34	23.75	20.47	19.18	20.35	19.47	18.36	15.54	14.66
Control	26.94	24.04	22.51	24.09	22.53	22.83	21.57	22.42	22.78

Table S2. Stress scores (PSS) across eight weeks for the experimental and control groups.

Group	Pretest	Week 1	Week 2	Week 3	Week 4	Week 5	Week 6	Week 7	Week 8
Experimental	22.26	21.71	20.16	19.61	20.00	19.84	19.00	18.10	17.10
Control	-	21.86	21.44	21.81	21.64	21.47	20.64	21.22	21.14

Table S3. Life satisfaction scores (SWLS) across eight weeks for the experimental and control groups.

Group	Pretest	Week 1	Week 2	Week 3	Week 4	Week 5	Week 6	Week 7	Week 8
Experimental	17.42	18.00	18.58	19.03	18.71	18.10	18.16	18.03	18.81
Control	-	16.56	16.31	16.97	16.94	16.92	17.25	16.72	16.75
